# Comparative Evaluation of the Effectiveness of At-Home Dental Bleaching Using Different Concentrations of Carbamide Peroxide for Different Time Intervals: A Randomized Clinical Trial

**DOI:** 10.7759/cureus.115502

**Published:** 2026-08-31

**Authors:** Nandani Kumari, Surendra Kumar Mishra, Syed Mukhtar-un-Nisar Andrabi, Aparna Rai, Akash Ganguly, Chandani Agrawal, Aradhana Singh

**Affiliations:** 1 Conservative Dentistry and Endodontics, Faculty of Dental Sciences, Banaras Hindu University, Varanasi, IND; 2 Conservative Dentistry and Endodontics, Dr. Ziauddin Ahmad Dental College and Hospital, Aligarh Muslim University, Aligarh, IND; 3 Oral and Maxillofacial Surgery, Post Graduate Institute of Dental Education and Research, Indira Gandhi Institute of Medical Sciences, Patna, IND

**Keywords:** at-home bleaching, carbamide peroxide, randomized clinical trial, tooth bleaching, tooth sensitivity, vita shade guide

## Abstract

Background

This randomized clinical trial evaluated the efficacy and safety of three concentrations of carbamide peroxide (CP) (10%, 22%, and 35%) for at-home tooth bleaching with reduced application times for the higher concentrations, comparing color change and tooth sensitivity to determine whether higher CP concentrations applied for shorter durations achieve equivalent or superior whitening outcomes without increasing patient discomfort. This study aimed to evaluate whether in adults aged 18-60 years with a baseline shade of A2 or darker (Population), does 22% or 35% CP for 30 minutes/day (Intervention), compared with 10% CP for four hours/day (Comparison), produce equivalent or superior tooth color change without increased sensitivity (Outcome) over three weeks with one-month follow-up.

Methodology

A total of 84 participants were randomly allocated 1:1:1 to Group I (10% CP, four hours/day), Group II (22% CP, 30 minutes/day), and Group III (35% CP, 30 minutes/day) for three weeks. Tooth color was assessed at baseline, weekly, and at one-month follow-up using VITA Classical and VITA Bleachedguide 3D-MASTER shade guides by a single blinded, calibrated evaluator. Tooth sensitivity was recorded daily using a Visual Analog Scale. Two-way repeated-measures analysis of variance with Tukey’s HSD post-hoc test was used for color data; Kruskal-Wallis test was used for sensitivity data. Significance was set at a p-value of 0.05; pairwise mean differences with 95% confidence intervals (CIs) and Cohen’s d were additionally computed.

Results

All 84 randomized participants (28 per group) completed the study with no losses to follow-up. All groups showed significant color improvement from baseline (p < 0.001). Groups II and III whitened faster than Group I during weeks one to two (mean difference = 1.7-2.2 SGU; Cohen’s d > 0.7); Group II converged with Group I by week three, while Group III remained significantly higher through one month. Sensitivity was mild in all groups with no significant intergroup differences at any time point (p > 0.05). Sensitivity occurred in 9/28 (32%) of Group I, 13/28 (45%) of Group II, and 15/28 (53.5%) of Group III; relative risk versus Group I was 1.44 (95% CI = 0.74-2.82) for Group II and 1.67 (95% CI = 0.88-3.16) for Group III, neither of which was significant.

Conclusions

Higher CP concentrations (22% and 35%), applied for 30 minutes/day, produced faster initial whitening than conventional 10% CP for four hours/day and achieved comparable outcomes, without a statistically significant increase in tooth sensitivity, though wide CIs around the relative risk estimates mean a true dose-related difference cannot be excluded. These findings support 22% and 35% CP for 30 minutes/day as effective, time-efficient alternatives to the conventional protocol, pending confirmation with spectrophotometric measurement, patient-reported outcomes, and longer follow-up.

## Introduction

The increasing emphasis on smile esthetics has led to significant advancements in esthetic dentistry, making tooth whitening one of the most sought-after minimally invasive dental procedures [1,2]. Innovations in bleaching products and delivery methods have expanded options from professional in-office treatments to convenient at-home systems, improving accessibility without compromising safety [3].

Contemporary bleaching systems employ hydrogen peroxide (HP) or carbamide peroxide (CP) as active ingredients. These agents decompose to generate free radicals that oxidize chromogenic molecules embedded within enamel and dentin, producing the characteristic whitening effect. The efficiency of oxidation is governed by the concentration of the bleaching agent, the duration of contact with the tooth surface, and the chemical environment at the enamel interface [4,5].

Carbamide peroxide is a stable compound that decomposes slowly into HP and urea in the presence of water. On dental surfaces, CP releases HP at approximately one-third of its nominal concentration, meaning 10% CP yields approximately 3.35% HP. This slower release rate, relative to HP-based products, contributes to its established safety profile for at-home use [5,6].

CP formulations approved for at-home use are typically available in concentrations of 10% to 35% [7]. The 10% CP concentration, applied for approximately four hours daily over three weeks using custom-fitted trays, has historically been the gold standard, with well-documented efficacy and safety profiles [8-12].

Despite its effectiveness, prolonged daily tray use presents compliance challenges [13,14], prompting interest in higher-concentration CP formulations (22-35%) for shorter 30-minute daily applications. da Costa et al. [15] found comparable whitening and tooth sensitivity with 35% CP and 14% HP applied for 30 minutes daily, while Sutil et al. [16] reported equivalent whitening with 37% CP for 30 minutes versus 10% CP for four hours daily, without increased sensitivity or gingival irritation. However, evidence comparing a broader range of CP concentrations using standardized short application times remains limited.

Tooth sensitivity remains the most frequently reported adverse effect of peroxide-based bleaching, particularly with high-concentration formulations [17]. Current evidence suggests that shortening application time may mitigate sensitivity while preserving efficacy, but robust comparative data across concentration ranges are lacking [15-17].

Accordingly, three research hypotheses were formulated for the present trial. First, higher concentrations of carbamide peroxide (22% and 35%), each applied for a shorter 30-minute daily duration, would produce color improvement comparable to or greater than that of 10% CP applied for four hours/day over the three-week treatment period. Second, higher concentrations of CP applied for the shorter 30-minute daily duration would not significantly increase tooth sensitivity compared with the conventional four-hour/day, 10% CP protocol. Third, the rate of color change would differ across the three-week treatment period between groups, with the higher-concentration, shorter-duration groups achieving earlier, more pronounced whitening in the first two weeks despite their much shorter daily contact time.

These hypotheses can be consolidated into a single primary objective, expressed in PICO format: to compare, in adults with a baseline shade of A2 or darker undergoing at-home bleaching (Population), 22% and 35% CP applied for 30 minutes/day (Intervention) against 10% CP applied for four hours/day (Comparison), on tooth color change and tooth sensitivity over three weeks of treatment and one month of follow-up (Outcome). The three concentrations were selected to represent, respectively, the established gold-standard protocol (10% CP), an intermediate at-home formulation (22% CP), and the upper limit of the approved at-home range (35% CP), allowing efficacy and safety to be compared across the full clinically relevant concentration spectrum under a uniform, abbreviated exposure time for the two higher-concentration arms. The primary outcome was change in tooth color/shade from baseline, assessed using validated shade guides. The secondary outcome was patient-reported tooth sensitivity, recorded daily throughout the treatment period.

## Materials and methods

Ethics approval and trial registration

This clinical trial was approved by the Institutional Ethics Committee, Faculty of Medicine, Aligarh Muslim University (AMU), Aligarh, India (approval number: IECJNMC/923), and was prospectively registered in the Clinical Trials Registry-India (CTRI) (registration number: CTRI/2024/03/064772). The study was conducted in accordance with the Declaration of Helsinki, and written informed consent was obtained from all participants. The primary outcome was change in tooth color/shade, and the secondary outcome was tooth sensitivity.

Study design and setting

This was a single-blind, parallel-group, three-arm randomized clinical trial conducted in accordance with the CONSORT 2010 statement. The trial was conducted from August 2022 to August 2024 at the postgraduate clinic of the Department of Conservative Dentistry and Endodontics, Dr. Ziauddin Ahmad Dental College and Hospital, Aligarh Muslim University, Aligarh, India.

Blinding

Given the inherent differences in tray-wear duration between groups, complete blinding of participants and the treating operator was not feasible. However, color assessments were performed by a single calibrated evaluator who was blinded to group allocation and had no role in randomization or treatment administration.

Sample size calculation

Sample size was calculated a priori using G*Power (version 3.1.9.7) based on a one-way analysis of variance (ANOVA) model, an anticipated effect size (f) of 0.39, alpha of 0.05, and power of 80%, yielding a minimum requirement of 25 participants per group. To account for an anticipated 10% attrition, 28 participants per group were enrolled, giving a total sample size of 84 (N = 84).

Participant recruitment and eligibility

Patients presenting to the outpatient department (OPD) of Conservative Dentistry and Endodontics seeking teeth-whitening treatment, supplemented by print and social media advertisements of the service, were screened for eligibility. The total number of patients screened for eligibility and the number excluded before randomization were not formally recorded.

Inclusion Criteria

Age 18-60 years, good general and oral health, all maxillary anterior teeth free from carious or non-carious cervical lesions, absence of active periodontal disease, and a minimum baseline shade of A2 (or darker) on the VITA Classical shade guide.

Exclusion Criteria

A history of tooth sensitivity, prior professional or at-home tooth whitening, severe intrinsic discoloration, visible enamel cracks, current pregnancy or breastfeeding, and active tobacco smoking.

Randomization and allocation concealment

A total of 84 eligible participants were randomly allocated to one of three groups in a 1:1:1 ratio, with 28 participants assigned to each group. The randomization sequence was prepared by an independent research assistant who was not involved in participant recruitment or treatment. The allocation sequence consisted of an equal number of assignments to each of the three groups (28 assignments per group), which were arranged in a random order. Each allocation was placed in a sequentially numbered, sealed, opaque envelope. The envelopes were kept securely by the independent research assistant and were opened sequentially only after the participant had provided written informed consent. Thus, the treating operator remained unaware of the group assignment until after enrolment and consent, thereby ensuring allocation concealment.

Participants were allocated to the following three groups: Group I received 10% CP gel (Opalescence®️ 10%, Ultradent Products Inc., South Jordan, UT, USA), applied for four hours/day (n = 28); Group II received 22% CP gel (Opalescence®️ 22%, Ultradent Products Inc.), applied for 30 minutes/day (n = 28); and Group III received 35% CP gel (Opalescence®️ 35%, Ultradent Products Inc.), applied for 30 minutes/day (n = 28).

Rationale for concentration and duration selection

The 10% CP group served as the comparator, representing the conventional gold-standard concentration and application duration (four hours/day). The 22% CP concentration represents an intermediate formulation within the approved at-home range. The 35% CP concentration was included to explore the efficacy and safety of a higher-concentration, shorter-duration protocol. The 22% and 35% CP groups were both prescribed the same 30-minute daily application duration, as this represents the manufacturer-recommended minimum application time for these higher-concentration formulations and mirrors protocols investigated previously [15,16]. Comparing each higher concentration against the conventional four-hour, 10% CP regimen allows concentration and duration to be evaluated together as a combined, clinically relevant alternative to the standard protocol.

Clinical procedures

Two weeks before bleaching, all participants underwent professional oral prophylaxis, including supragingival scaling with an ultrasonic scaler and polishing of buccal/labial surfaces with prophylaxis paste (Ammdent Prolax, Ammdent, Punjab, India) using a rubber cup on a low-speed handpiece.

Alginate impressions (Tropicalgin®, Zhermack, Italy) were recorded for the maxillary and mandibular arches. Stone casts were poured, and custom vinyl-acetate bleaching trays (2 mm thickness) were fabricated on a vacuum-forming unit and trimmed to a 2 mm scalloped margin at the gingival edge.

Participants were instructed on the application of the bleaching material and directed to use the tray containing the corresponding CP concentration for specified time intervals as per their assigned groups (i.e., 10% CP for four hours/day, 22% CP for 30 minutes/day, and 35% CP for 30 minutes/day). Each treatment lasted for three weeks. Participants brushed twice daily with a fluoridated, non-bleaching toothpaste and were instructed not to use desensitizing agents throughout the study.

Compliance Monitoring and Dietary Restrictions

Daily compliance with the assigned bleaching protocol was monitored using a self-reported patient diary, in which participants recorded the time of gel application and duration of tray wear each day; diaries were reviewed by the treating operator at each weekly follow-up visit. Participants were additionally instructed to avoid known staining substances, including tea, coffee, red wine, cola, and tobacco products, for the duration of the three-week treatment period, and adherence to this restriction was verbally confirmed at each weekly visit.

Outcome measures

For assessing color change (primary outcome), tooth shade was assessed at baseline, at the end of weeks one, two, and three, and at one month post-treatment by the same blinded, calibrated evaluator under standardized natural light, using both the VITA Classical and VITA Bleachedguide 3D-MASTER shade guides. Shade scores were converted to numerical values (1-16 for VITA Classical; 1-15 for Bleachedguide) per Paravina [18], expressed in shade guide units (SGUs). VITA Bleachedguide 3D-MASTER was designated the primary outcome measure for all analyses reported here, because its extended range of lighter shade tabs gives finer discrimination in the value range most relevant to tracking bleaching progress [18]. VITA Classical scores were recorded at every assessment as a secondary/concordance check but are not reported further in this analysis.

For each participant, the daily Visual Analog Scale (VAS) scores recorded within a given week were summarized as that participant’s weekly sensitivity score using the median of that week’s daily values. These weekly per-participant medians were the unit of analysis for all between-group comparisons of tooth sensitivity.

Calibration of the evaluator

The evaluator underwent standardized training in shade-guide assessment before the study. Intra-rater reliability, assessed via two independent shade evaluations 48 hours apart, achieved a concordance correlation coefficient of ≥0.90 before study commencement.

Statistical analysis

Data were analyzed using SPSS Statistics version 26.0 (IBM Corp., Armonk, NY, USA). Normality was assessed with the Shapiro-Wilk test and Q-Q plots. Baseline comparability was verified using one-way ANOVA. Longitudinal shade-score changes were analyzed with two-way repeated-measures ANOVA (factors: time and concentration group) followed by Tukey’s honestly significant difference (HSD) post-hoc test. Tooth sensitivity (VAS) was summarized per participant per week as the median of that week’s daily scores (see Outcome Measures); being non-normally distributed, it was compared with the Kruskal-Wallis test, and the proportion of participants reporting any sensitivity was compared using Pearson’s chi-square test and relative risk with a 95% confidence interval (CI). Significance was set at p-values of 0.05. Throughout the Results section, the relevant test statistic (F, H, or χ²) and the name of the statistical test are reported alongside every p-value.

Pairwise mean differences between groups at each time point were additionally quantified with 95% CIs and Cohen’s d effect sizes, computed directly from the reported group means, standard deviations, and sample sizes (independent-samples approach; these results should be read as a descriptive supplement to the repeated-measures ANOVA/Tukey HSD inferential analysis).

## Results

Participant flow

Of the 84 participants randomized (28 per group), all 84 completed the full three-week treatment protocol and the one-month follow-up. There were no losses to follow-up and no exclusions, so the analyzed sample equals the randomized sample in every group (n = 28/28, 100% retention in each arm) (Figure 1).

**Figure 1:**
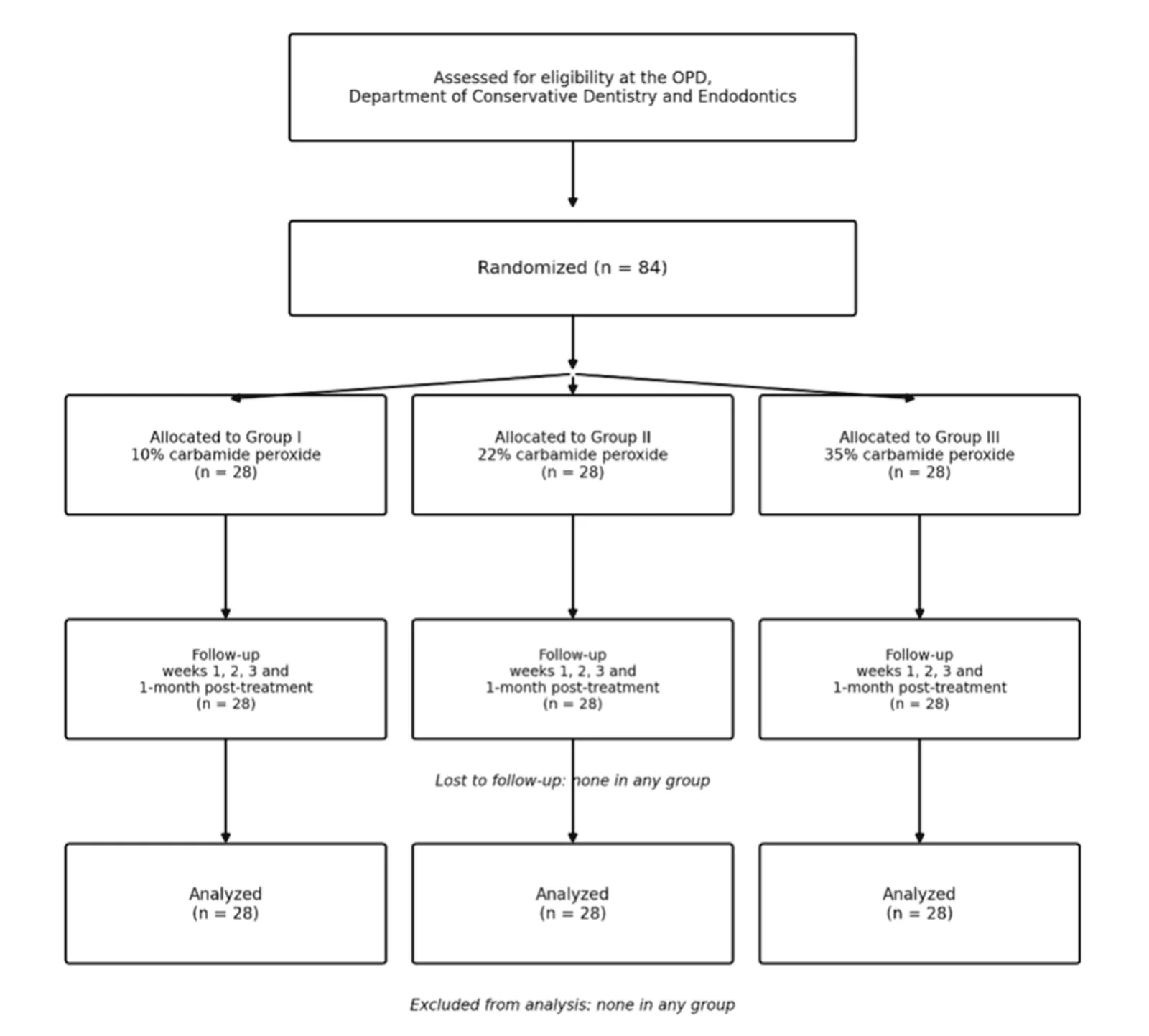
CONSORT participant flow diagram.

Baseline characteristics

The mean age was 26.0 ± 5.67 years in Group I, 25.6 ± 4.31 years in Group II, and 23.4 ± 4.86 years in Group III, with no statistically significant between-group difference (one-way ANOVA, F(2,81) = 2.37, p = 0.10). Overall, 15 of 28 participants (53.6%) in Group I, 17/28 (60.7%) in Group II, and 16/28 (57.1%) in Group III were female, with no significant between-group difference in sex distribution (Pearson’s chi-square test, χ²(2) = 0.29, p = 0.86). One-way ANOVA confirmed no significant between-group difference in baseline canine shade score (F(2,81) = 1.12, p = 0.33) (Table 1).

**Table 1:** Baseline demographic and clinical characteristics. *: One-way analysis of variance (age, baseline shade) or Pearson’s chi-square test (sex distribution). CP: carbamide peroxide; SD: standard deviation; SGU: shade guide unit

Characteristic	Group I (10% CP), n = 28	Group II (22% CP), n = 28	Group III (35% CP), n = 28	Test statistic, p-value*
Age, years (mean ± SD)	26.0 ± 5.67	25.6 ± 4.31	23.4 ± 4.86	F(2,81) = 2.37, p = 0.10
Female, n (%)	15 (53.6)	17 (60.7)	16 (57.1)	χ²(2) = 0.29, p = 0.86
Baseline canine shade (SGU, mean ± SD)	5.2 ± 1.1	5.1 ± 1.2	5.0 ± 1.0	F(2,81) = 1.12, p = 0.33

Color change assessment

Two-way repeated-measures ANOVA revealed a statistically significant interaction between time and CP concentration for the primary outcome measure, VITA Bleachedguide 3D-MASTER (p < 0.001); results are interpreted primarily through this interaction, consistent with standard two-way ANOVA convention. Findings using VITA Classical, recorded concurrently, were directionally consistent and are not reported in detail.

Within-group comparisons

All three groups showed statistically significant, progressive increases in canine shade score across successive assessments (Tukey’s HSD, p < 0.001 for each within-group comparison), indicating consistent whitening within each group through the one-month follow-up (Table 2, Figure 2).

**Table 2:** Canine shade score (SGU, VITA Bleachedguide 3D-MASTER) by group and time point. *: Two-way repeated-measures analysis of variance with Tukey’s honestly significant difference post hoc test at each time point. Values are mean ± SD (shade guide units). NS: not significant (p > 0.05). SGU: shade guide unit

Time point	Group I (10% CP)	Group II (22% CP)	Group III (35% CP)	Test statistic, p-value*	Significance
Baseline	5.2 ± 1.1	5.1 ± 1.2	5.0 ± 1.0	F(2,81) = 1.12, p = 0.33	NS
Week 1	7.4 ± 1.3	9.1 ± 1.5	9.3 ± 1.4	F(2,81) = 15.52, p < 0.001	II, III > I
Week 2	9.6 ± 1.4	11.5 ± 1.6	11.8 ± 1.5	F(2,81) = 17.66, p < 0.001	II, III > I
Week 3	11.2 ± 1.5	12.3 ± 1.4	12.9 ± 1.3	F(2,81) = 7.53, p = 0.001	III > I
1 month	10.9 ± 1.6	12.0 ± 1.5	12.5 ± 1.4	F(2,81) = 4.50, p = 0.014	III > I

**Figure 2:**
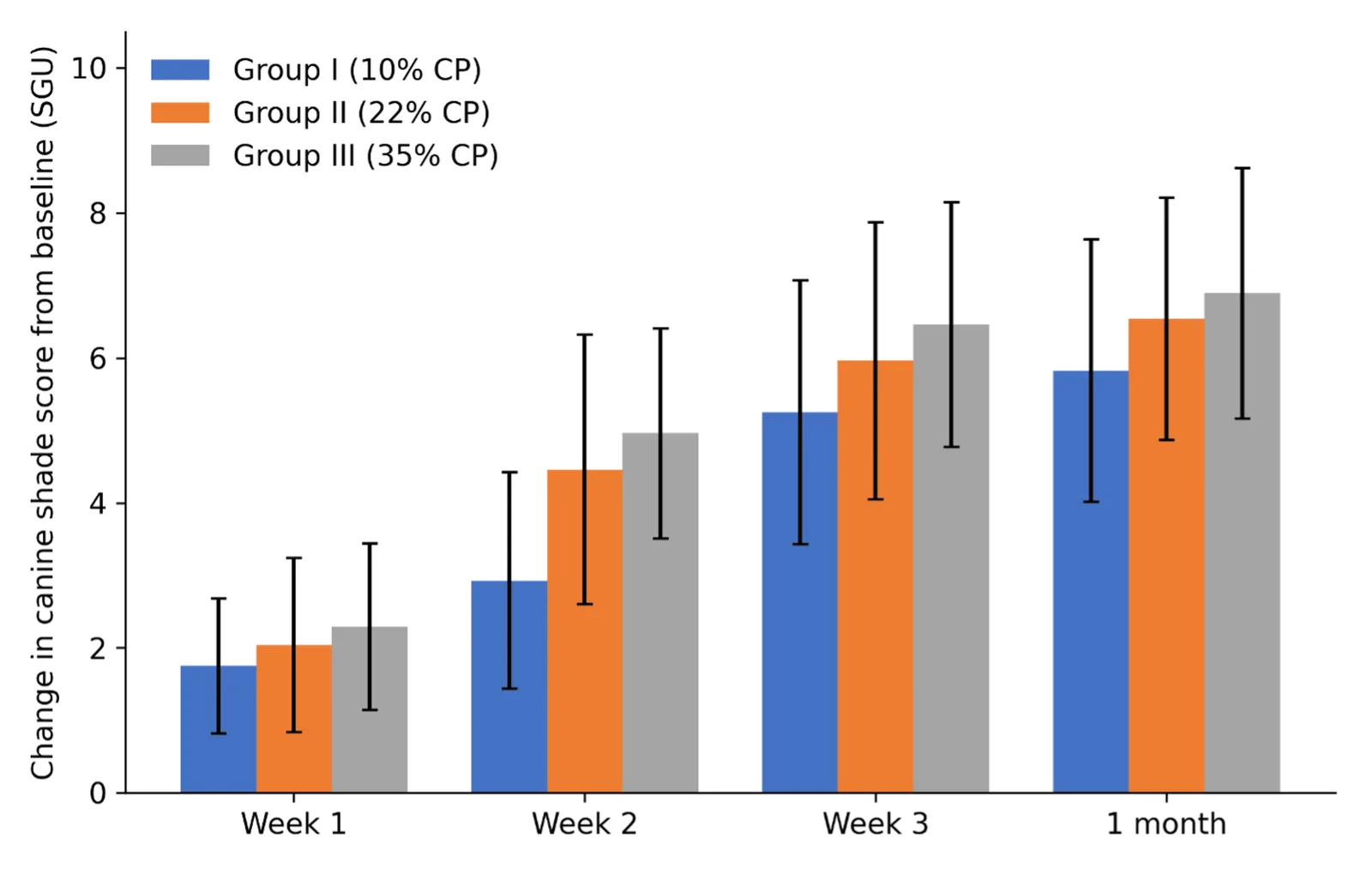
Change in canine shade score from baseline (mean ± SD, SGUs) by group and time point. CP: carbamide peroxide; SGU: shade guide unit

Between-group comparisons

During weeks one and two, Group I showed significantly lower cumulative shade change than Groups II and III (Tukey’s HSD; week 1: p = 0.001; week 2: p < 0.001); Groups II and III did not differ significantly at either time point. By week three, Group I remained significantly lower than Group III only (p = 0.001). At the one-month follow-up, Group I remained lower than Group III (p = 0.014), while Groups II and III remained comparable (Table 3).

**Table 3:** Between-group comparisons of canine shade change (Tukey’s honestly significant difference) using VITA Bleachedguide 3D-MASTER. *: Tukey’s honestly significant difference post hoc pairwise comparisons following two-way repeated-measures analysis of variance. Significance threshold: p = 0.05. An independent-samples t-equivalent, derived directly from the mean differences and 95% confidence intervals already reported for these same comparisons, is provided in Table 4.

Time point	I vs II, p (Tukey’s HSD)*	I vs III, p (Tukey’s HSD)*	II vs III, p (Tukey’s HSD)*	Summary
Week 1	0.001	0.001	0.98	I < II = III
Week 2	<0.001	<0.001	0.87	I < II = III
Week 3	0.06	0.001	0.73	I ≈ II < III
1 month	0.09	0.014	0.81	I ≈ II; I < III

Effect-size analysis

Pairwise mean differences in canine shade score were computed directly from the descriptive statistics in Table 2, together with 95% CI, an independent-samples t-statistic (df = 54) derived from that same CI, and Cohen’s d (Table 4). Differences between Group I and Groups II/III were large in magnitude at weeks one to two (d ≈ 0.8-1.5) and moderate to large at week three and one month, while differences between Groups II and III were consistently small and non-significant (d ≈ 0.1-0.4), mirroring the Tukey HSD pattern in Table 3.

**Table 4:** Pairwise mean differences, 95% CI, independent-samples t-statistic, and Cohen’s d for canine shade score (VITA Bleachedguide 3D-MASTER). *: Independent-samples t-test (two-tailed, df = 54), with t and p derived directly from the mean difference and 95% CI reported in the adjacent columns; presented as a descriptive supplement to, not a replacement for, the repeated-measures analysis of variance/Tukey’s honestly significant difference analysis in Tables 2, 3. Negative mean differences indicate the first-listed group had a lower shade score (less whitening). Cohen’s d: ~0.2 small, ~0.5 medium, ~0.8 large. SGU: shade guide unit; CI: confidence interval

Time point	Comparison	Mean difference (SGU)	95% CI	t (df = 54)*	P-value*	Cohen’s d
Week 1	I vs. II	-1.70	-2.45 to -0.95	-4.54	<0.001	-1.21
	I vs. III	-1.90	-2.62 to -1.18	-5.29	<0.001	-1.41
	II vs. III	-0.20	-0.98 to 0.58	-0.51	0.61	-0.14
Week 2	I vs. II	-1.90	-2.71 to -1.09	-4.70	<0.001	-1.26
	I vs. III	-2.20	-2.98 to -1.42	-5.65	<0.001	-1.52
	II vs. III	-0.30	-1.13 to 0.53	-0.72	0.47	-0.19
Week 3	I vs. II	-1.10	-1.88 to -0.32	-2.83	0.007	-0.76
	I vs. III	-1.70	-2.45 to -0.95	-4.54	<0.001	-1.21
	II vs. III	-0.60	-1.32 to 0.12	-1.67	0.10	-0.44
1 month	I vs. II	-1.10	-1.93 to -0.27	-2.66	0.010	-0.71
	I vs. III	-1.60	-2.41 to -0.79	-3.96	<0.001	-1.06
	II vs. III	-0.50	-1.28 to 0.28	-1.29	0.20	-0.34

Tooth sensitivity

Kruskal-Wallis testing showed no statistically significant differences in median VAS scores between groups at any week (week 1: H(2) = 2.69, p = 0.26; week 2: H(2) = 1.16, p = 0.56; week 3: H(2) = 1.02, p = 0.60); median scores were 0 in all groups at all time points, and most sensitivity was mild-to-moderate, peaking at week one and declining thereafter.

Overall, 9/28 participants (32%, 95% CI = 17.9-50.6%) in Group I, 13/28 (45%, 95% CI = 29.5-64.1%) in Group II, and 15/28 (53.5%, 95% CI = 35.8-70.4%) in Group III reported sensitivity on at least one occasion during treatment. Relative to Group I, the relative risk of reporting any sensitivity, tested with Pearson’s chi-square test, was 1.44 (95% CI = 0.74-2.82; χ²(1) = 1.20, p = 0.27) for Group II and 1.67 (95% CI = 0.88-3.16; χ²(1) = 2.62, p = 0.11) for Group III, with neither reaching statistical significance (Table 5, Figure 3).

**Table 5:** Frequency of tooth sensitivity, absolute risk, and relative risk (vs. Group I). *: Pearson’s chi-square test (uncorrected, df = 1), computed directly from the counts in this table. A participant was classified as “present” if sensitivity was reported on any follow-up occasion. CI: confidence interval; CP: carbamide peroxide

Group	Present, n	Absent, n	Absolute risk, % (95% CI)	Relative risk (95% CI)	χ²(1), p-value*
I (10% CP)	9	19	32.0 (17.9–50.6)	reference	—
II (22% CP)	13	15	45.0 (29.5–64.1)	1.44 (0.74–2.82)	1.20, 0.27
III (35% CP)	15	13	53.5 (35.8–70.4)	1.67 (0.88–3.16)	2.62, 0.11

**Figure 3:**
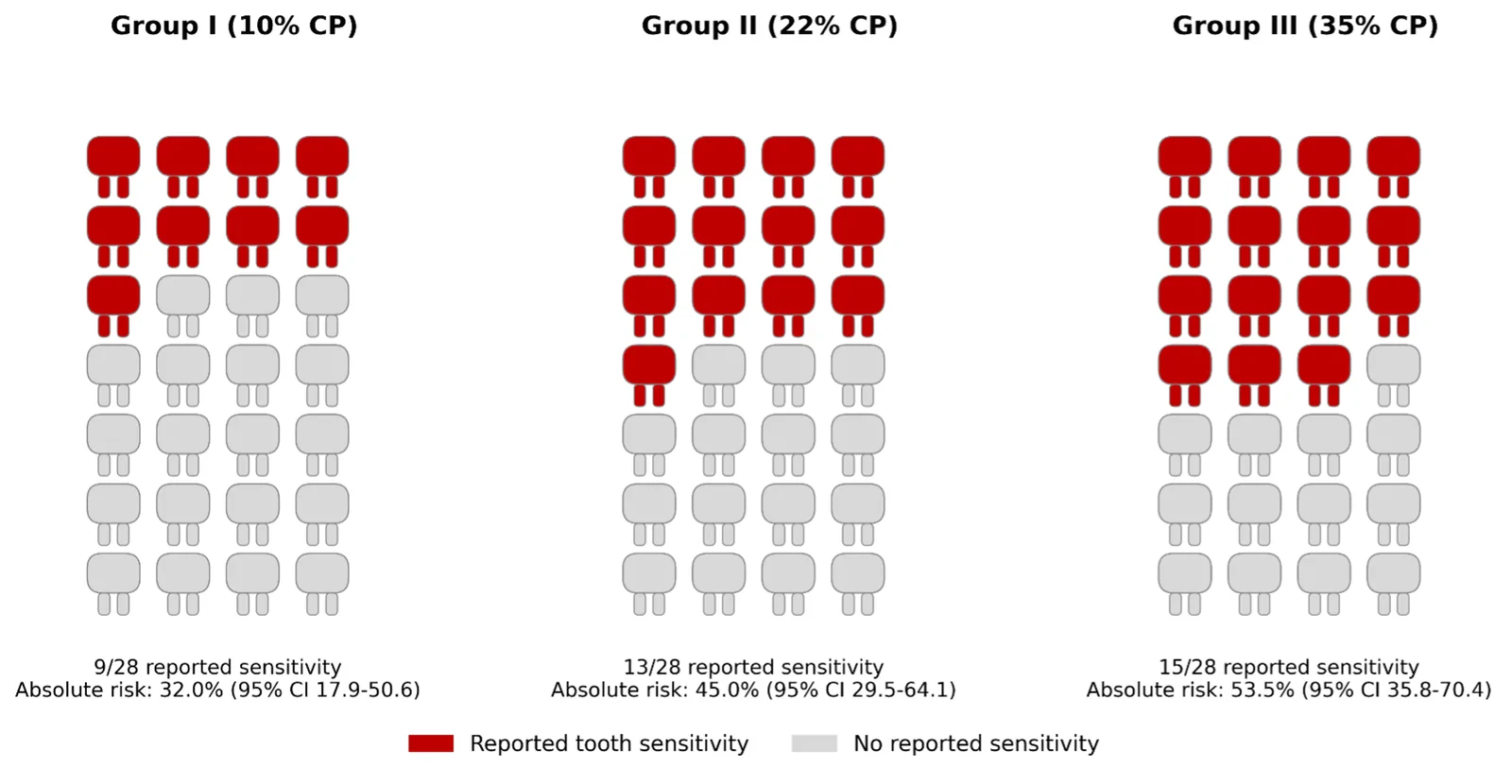
Frequency of participants reporting tooth sensitivity on at least one occasion, by group. CI: confidence interval; CP: carbamide peroxide

Representative clinical photographs

Representative baseline and post-treatment intraoral photographs for one participant from each treatment group are shown in Figures 4-6, illustrating the visually appreciable degree of whitening achieved with each protocol. These images are illustrative examples selected to represent typical outcomes within each group and were not used for quantitative or standardized assessment of whitening uniformity; any non-uniform or blotchy appearance visible in individual photographs was not systematically evaluated as a study outcome.

**Figure 4:**
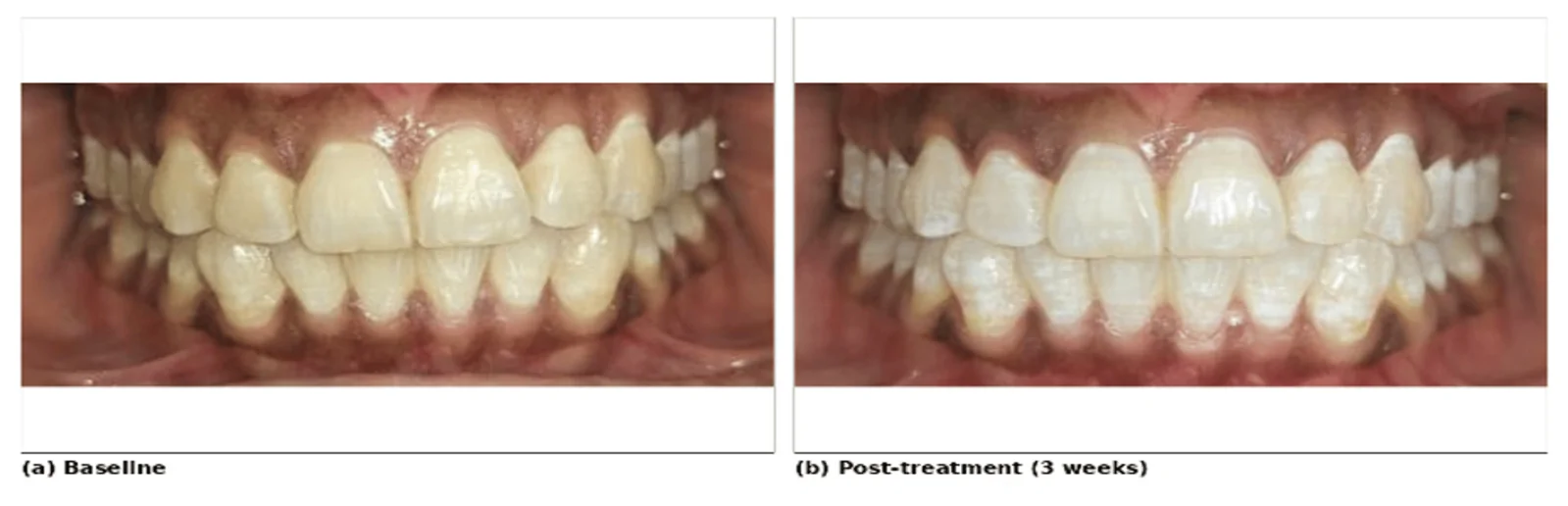
Representative clinical photographs, Group I (10% carbamide peroxide, four hours/day): (a) baseline and (b) post-treatment (end of week three).

**Figure 5:**
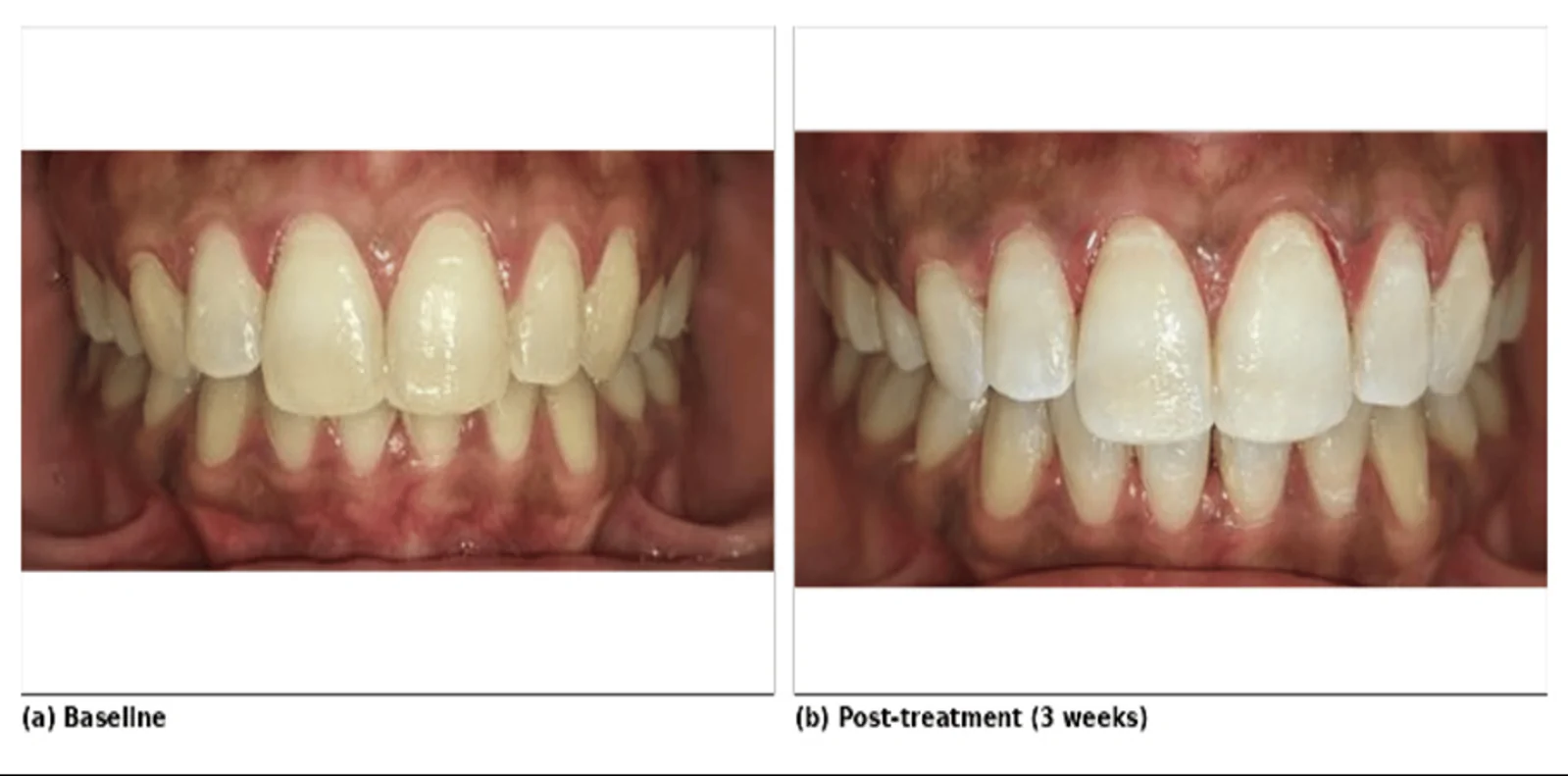
Representative clinical photographs, Group II (22% carbamide peroxide, 30 minutes/day): (a) baseline and (b) post-treatment (end of week three).

**Figure 6:**
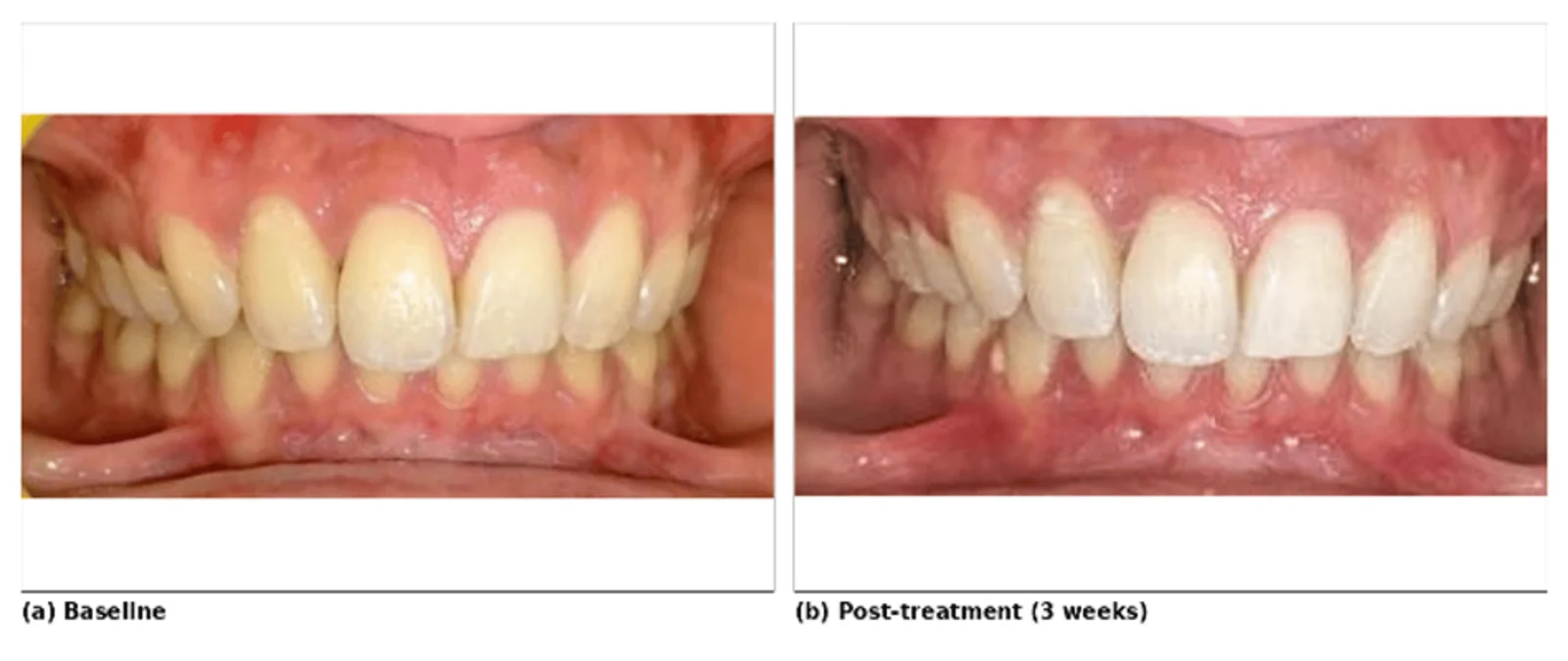
Representative clinical photographs, Group III (35% carbamide peroxide, 30 minutes/day): (a) baseline and (b) post-treatment (end of week three).

## Discussion

This randomized clinical trial compared three CP protocols: 10% CP for four hours/day, 22% CP for 30 minutes/day, and 35% CP for 30 minutes/day, each over three weeks. Higher-concentration, shorter-duration protocols (22% and 35% CP) produced more rapid whitening in the first two weeks; Group II converged with the conventional 10% CP protocol by week three, while Group III remained modestly but significantly ahead of Group I through the one-month follow-up. Importantly, this speed advantage was not accompanied by a statistically significant increase in tooth sensitivity.

The representative clinical photographs (Figures 4-6) show some visible non-uniformity in the whitening effect for individual participants. This is a recognized, transient phenomenon in peroxide-based bleaching, generally attributed to regional variation in enamel porosity, hydration, and initial chromogen distribution, and it is not unique to any single concentration or protocol in this study. However, whitening uniformity and other visible surface changes were not systematically assessed as a study outcome, and no standardized index of treatment-related complications (e.g., white-spot lesions, enamel translucency changes, or gingival irritation) was applied beyond the VAS sensitivity measure. Consequently, we are unable to report whether uniformity or other visible adverse effects differed systematically between groups, and this represents an important outcome for future trials to assess using standardized clinical photography protocols and objective grading criteria.

The 10% CP formulation, comprising approximately 3.5% HP and 6.5% urea, has been extensively validated as the gold-standard agent for at-home bleaching when applied for several hours daily [19,20]. The faster initial whitening observed in Groups II and III is mechanistically explained by the greater flux of reactive oxygen species generated per unit time at higher CP concentrations, which compensates for their much shorter daily contact time by accelerating oxidation of chromogenic compounds within enamel and dentine. Conversely, the lower radical production rate of 10% CP is offset by its considerably longer daily application (4 hours versus 30 minutes), explaining why Group I achieved comparable cumulative outcomes only by week three.

Although Groups II and III did not differ significantly from one another at any time point (Table 3), their trajectories relative to the conventional 10% CP protocol were not identical. Group II’s advantage over Group I was confined to weeks one and two and had resolved by week three, whereas Group III remained significantly ahead of Group I through the one-month follow-up. This suggests that, within the three-week treatment window and beyond, 35% CP may offer a more durable margin over the conventional protocol than 22% CP, even though the two higher-concentration arms were statistically indistinguishable from each other throughout. Whether this modest, sustained difference is large enough to be clinically meaningful to patients or clinicians is uncertain and was not assessed with a minimal clinically important difference (MCID) threshold in this trial. Given that 22% CP achieved comparable whitening to 35% CP with a numerically (though not statistically) lower rate of reported sensitivity (45.0% vs. 53.5%) (Table 5), clinicians and patients may reasonably favor 22% CP as the more conservative choice among the two higher-concentration options, reserving 35% CP for cases where the more sustained shade advantage through one month is a specific priority. Confirming whether this longer-term difference between 22% and 35% CP is clinically meaningful will require future trials incorporating a predefined MCID and longer follow-up.

An important methodological caveat is that concentration and application duration were varied simultaneously across groups rather than independently: Group I received a lower concentration for a longer duration, while Groups II and III received higher concentrations for a fixed, shorter duration. This design reflects real-world at-home bleaching protocols, in which concentration and duration are conventionally paired in this manner, but it also means the trial cannot disentangle the relative contribution of concentration versus exposure time to the observed differences in whitening rate. The faster early whitening in Groups II and III could reflect the higher CP concentration, the specific 30-minute exposure window, or their interaction, and the present design does not permit these explanations to be distinguished. A factorial design varying concentration and duration independently would be needed to isolate their individual contributions.

These findings are broadly consistent with those of Sutil et al. [16], who found no significant difference in final color outcomes between 10% and 37% CP after three weeks of at-home use, and with other work demonstrating cumulative equivalence between longer-duration, lower-concentration and shorter-duration, higher-concentration regimens.

The absence of a statistically significant increase in sensitivity with 35% CP despite its higher concentration is noteworthy. Higher peroxide concentrations can, in principle, induce transient pulp-cell irritation through diffusion of reactive oxygen species across dentin; however, limiting application of the higher-concentration gels to 30 minutes was not associated with a statistically detectable difference in sensitivity relative to the four-hour, 10% CP protocol in this sample. This is consistent with the findings of da Costa et al. [15] and Sutil et al. [16], who similarly observed that brief applications of high-concentration CP at home did not elevate sensitivity relative to conventional, longer-duration, lower-concentration protocols. This finding warrants cautious interpretation; however, the trial’s sample size was calculated a priori to detect differences in the primary outcome of tooth color change, not to establish non-inferiority or equivalence in tooth sensitivity, and the study was therefore underpowered to detect all but a large difference in adverse event rates. Consistent with this, the relative risk estimates (1.44 and 1.67 for 22% and 35% CP, respectively) were directionally suggestive of a dose-related trend but were statistically imprecise, with 95% CIs spanning both a reduced risk and an increase of up to nearly threefold. The absence of a significant difference should therefore be read as an absence of demonstrated difference rather than as evidence of equivalent safety.

The choice of subjective shade guide assessment, rather than spectrophotometric colorimetry, was guided by the validated methodology of Paravina [18] using the VITA Bleachedguide 3D-MASTER, which offers a granular range of lighter shades well-suited to tracking bleaching progress. Assessment of the canine as the sentinel tooth reflects its greater baseline chromacity and more pronounced post-bleaching color shift, rendering it a sensitive discriminator of bleaching efficacy. Nevertheless, incorporating objective spectrophotometric ΔE measurement alongside shade guide assessment in future trials would allow direct comparison with colorimetric standards and reduce the inter-observer subjectivity inherent to visual grading, even with a calibrated evaluator. Recent work using spectrophotometric CIELAB and Whiteness Index measurements to evaluate laser-assisted bleaching illustrates the objectivity and precision such instrumentation can bring to shade assessment, reinforcing the value of incorporating spectrophotometric methods alongside or instead of visual shade-guide grading in future trials [21].

Systematic reviews of at-home CP bleaching have confirmed the efficacy of tray-delivered CP across concentrations [7]. The one-month follow-up in the present study showed stable whitening across all groups, without clinically apparent color regression; however, this window remains short relative to the 6-12 month follow-up used in some comparable trials, and longer-term color stability and relapse cannot be excluded based on this study alone.

Limitations

Several limitations warrant consideration. Color change was assessed using visual shade guides rather than objective spectrophotometric methods, and whitening uniformity visible in clinical photographs was not systematically graded; future studies should incorporate spectrophotometric ΔE measurement. The one-month follow-up was insufficient to assess long-term color stability, and soft-tissue effects (e.g., gingival irritation) were not evaluated with a standardized index. As the sample size was calculated for the primary color change outcome only, the trial was not powered to test non-inferiority in tooth sensitivity. Because concentration and application duration were varied together by design, their independent contributions to whitening kinetics cannot be separated. Patient-reported satisfaction, compliance, and treatment preference were not collected prospectively. Finally, as a single-center trial recruited partly through service advertisement, with screening/exclusion numbers unrecorded, generalizability may be limited. Multicenter trials with objective color assessment, longer follow-up, and comprehensive safety and patient-reported outcomes are warranted.

## Conclusions

Within the limitations of this randomized clinical trial, the following conclusions are supported by the data. Higher concentrations of CP (22% and 35%), each applied for 30 minutes/day, produced faster initial whitening than the conventional 10% CP protocol applied for four hours/day, with statistically significant and large-magnitude differences at weeks one and two. By the end of week three, all three CP concentrations achieved comparable levels of tooth whitening, with no statistically significant difference between groups except for a residual difference between Group I and Group III. Tooth sensitivity was mild across all groups and did not differ significantly between them at any time point; relative-risk estimates suggested a possible dose-related trend that was not statistically significant and should be examined in an adequately powered future trial. Given the modest sample size (n = 28 per group) and the width of the 95% CIs around these relative risk estimates (0.74-2.82 for 22% CP; 0.88-3.16 for 35% CP), the absence of a statistically significant difference should be interpreted as an absence of demonstrated difference rather than as evidence that the three protocols carry equivalent sensitivity risk. Color stability at one month post-treatment was maintained in all groups without apparent rebound, though longer follow-up is needed to confirm durability. These conclusions apply specifically to the concentration-duration combinations investigated in this trial. Because concentration and application time were varied together rather than independently, the findings should not be interpreted as demonstrating an effect of concentration in isolation. Taken together, these findings support 22% and 35% CP applied for 30 minutes/day as effective and time-efficient alternatives to the conventional four-hour/day, 10% CP protocol for at-home tooth bleaching, pending confirmation with objective color measurement, patient-reported outcomes, and extended follow-up.
